# Supplementary figures and images for: Key role of Extracellular RNA in hypoxic stress induced myocardial injury
Source: PLoS One. 2021 Dec 9;16(12):e0260835. doi: 10.1371/journal.pone.0260835 (PMC8659422; doi:10.1371/journal.pone.0260835)

Supplementary Figure 1:

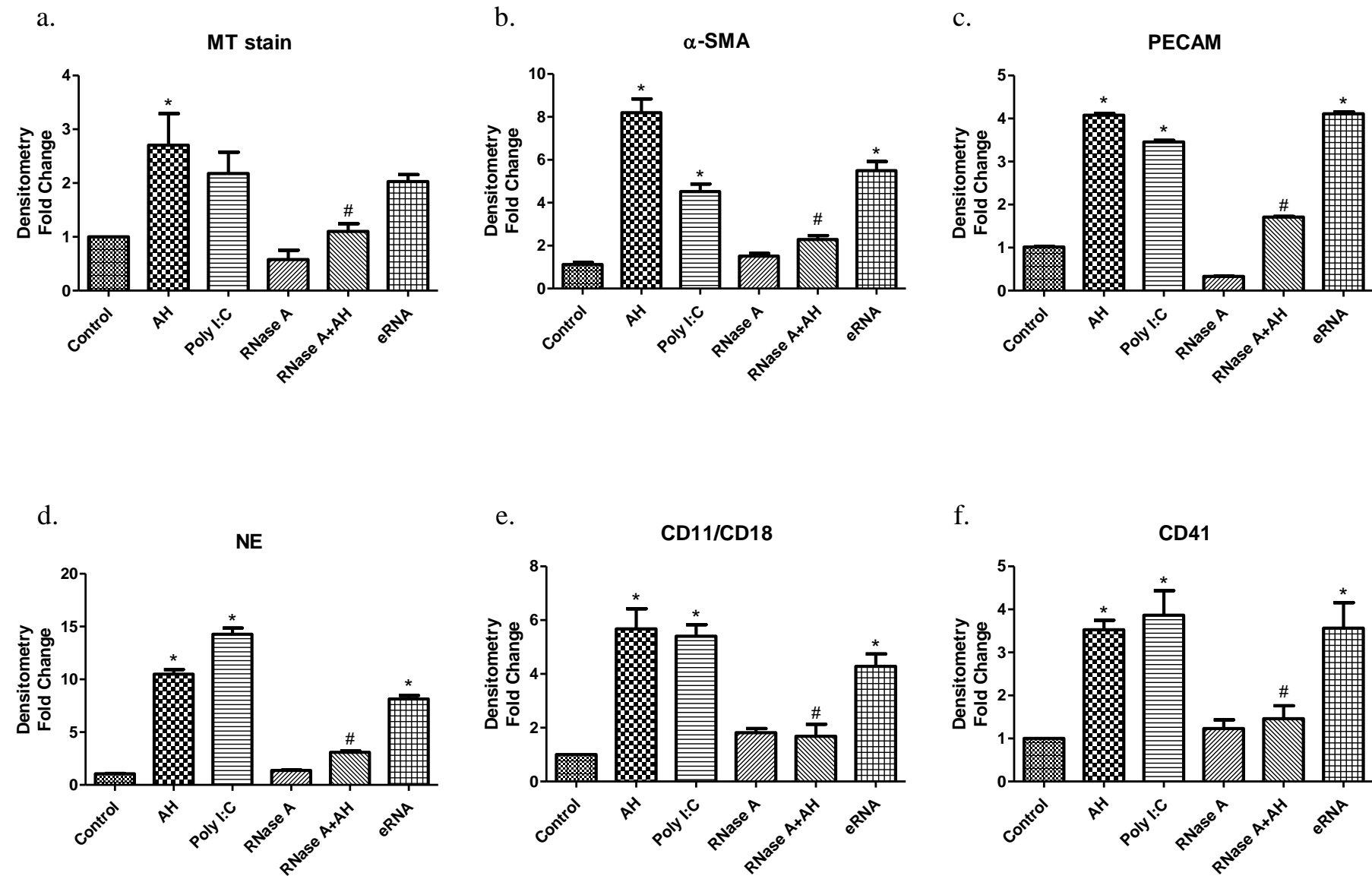

Supplement: S1 Fig — Densitometry analysis of (a) MT staining depicting collagen, (b) immunohistochemistry for α-SMA, (c) immunohistochemistry for PECAM, (d) immunohistochemistry for NE, (e) immunofluorescence for CD11/CD18 and (f) immunofluorescence for CD41. Data are shown as representative of three independent experiments, all performed in triplicate. One-way ANOVA revealed statistical significance in the results (*p< 0.05 groups w.r.t control; #p<0.05 groups w.r.t AH). (PDF) [file pone.0260835.s001.pdf]

Supplementary Figure 2:

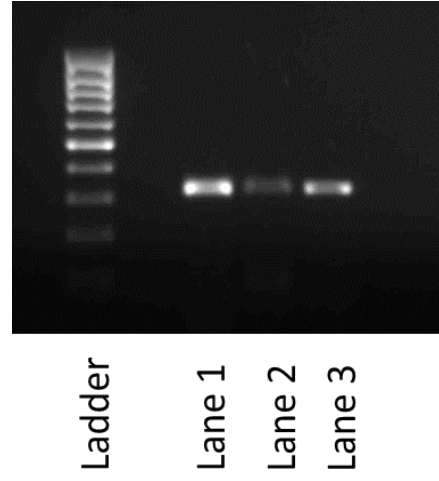

Supplement: S2 Fig — Lane 1- Control (no siRNA treatment), Lane 2- TLR3 siRNA treatment and Lane 3- non-specific siRNA treatment. (PDF) [file pone.0260835.s002.pdf]
