# Supplementary material for: Key role of Extracellular RNA in hypoxic stress induced myocardial injury
Source: PLoS One. 2021 Dec 9;16(12):e0260835. doi: 10.1371/journal.pone.0260835 (PMC8659422; doi:10.1371/journal.pone.0260835)
Supplement: Attachment — Submitted filename: Review Comments to the Author.docx [file pone.0260835.s003.docx]

**Review Comments to the Author**

Authors have studied the key role for eRNA and TLR3 in hypoxia-induced myocardial ischemic injury. The authors presented the mechanism of cTrop-T release and progression of acute myocardial infarction through TLR3-caspase -3 pathway in a murine model of acute hypoxia. The proposed aim and choice of testing the eNA is novel and has current interest in the field of cardiovascular biology. Although there is data provided in support of the aim and objectives, I have some concerns with the manuscript, primarily with methodology and some aspects where statements appear to confuse cause and effect. The paper will be of significant interest, provided the issues below are addressed and modifications are made.

**Major comments:**

1. The background should clearly state the purpose of the study.
2. In the abstract section, (page 2, line 25) the sentence, “Although the diagnosis is based on……” is incomplete.
3. Details of methodology are missing in several places.
   a. How long were these animals exposed to acute hypoxia conditions?
   b. In the method section, authors mentioned that 80ug of eRNA and eDNA were injected into the animal by intravenously through the tail vein. What is the starting material for isolating eRNA and eDNA from plasma? What is the yield eRNA and eDNA isolated from plasma of exposed animal?
   c. Is eDNA and eRNA from each animal or is it from pooled samples of exposed animals? Please give addition details in methods section.
   d. The methodology for isolating eDNA reported in the manuscript is missing. Please add this to methodology.
   e. In addition, the time point for the proposed experiments are not described. Please provide specific time point for histological and immunochemical study. Is the time point the same for all parameters?
   f. Under the methodology section, line 142, ELISA, the authors should provide detailed information about specific antibodies (primary and secondary antibody) and the dilution used for the assay. Methods do not have proper references and are not explained in detail.
   g. For Immunofluorescence, please detail antibody information for both primary and secondary antibodies for CD31, CD11/CD18, neutrophil elastase etc.
   h. Please provide higher magnification image showing leukocyte infiltration along with low magnification images.

**Minor comments**

4. In page 7, line 143 50ug plasma, it should be in uL
5. The quality of the Figure 1-4 is poor. Please maintain the same consistency in numbering all figure panels.
6. The manuscript needs critical editing for language and syntax errors.

Results from the present study agree with previously published reports. The above issues do not abrogate the potential importance of the paper; however, I do think it would be very interesting to see the modified manuscript including the answers for these queries.
