# Supplementary material for: Key role of Extracellular RNA in hypoxic stress induced myocardial injury
Source: PLoS One. 2021 Dec 9;16(12):e0260835. doi: 10.1371/journal.pone.0260835 (PMC8659422; doi:10.1371/journal.pone.0260835)
Supplement: Attachment — Submitted filename: response to reviewer.docx [file pone.0260835.s005.docx]

**Response to Reviewer-2:**

**Second Revision – author’s’ response	7^th^ Nov., 2021**

**All editing in the manuscript is presented as track change yellow.**

I am indebted to you for the valuable comments for overall improvement of the manuscript. Accordingly, in this revised paper, I give due weightage to comments and the resultant responses are:

**The answer to the Reviewer # 2**

**Major Comments:**

**Point: 1**

In Figure 1, quantitation of the eDNA, eRNA as well as SI markers were requested. While the quantitation of eDNA and eRNA were provided in the response letter, the plots were not changed to reflect quantitation. The authors fail to realize the significance of adding quantitation to the graphs in Figure 1. The intent is not to convince the reviewer but the reader. These numbers will serve as a guide to investigators in the field who may be doing similar experiments. The justification for not providing these numbers since it is a basic science study and not a clinical study is baseless and without any merit. In similar basic science studies, the authors have themselves published concentrations of eRNA and eDNA (Refs 1and 13 of the manuscript). They have also published quantitative values for ELISA in these publications that they have authored. Interestingly, the y-axis values in Figure 1a and 1b correspond to the values provided in the response letter and are not ‘fold changes’ as indicated in the plots.

**Author Response:** I wish to thank reviewer for the comments. We have addressed the comment of the reviewer and provided the bar graphs for quantitative analysis of all the SI markers (Figure 1 (c-g). Specific quantitative ELISA kits were used for this purpose. The plots for eRNA and eDNA have been corrected to represent the quantitation details.

**Point: 2**

The supplementary figures 1a, 1b and 1c should be shown alongside Figure 5(I) to bring more clarity to the changes. The arrows in the lower and higher magnification images do not match and sometimes they show different regions (Supp Fig 1a: Arrows in Poly I:C and RNase A+AH pairs; Supp Fig 1b: Arrows in Poly I:C and RNase A+AH pairs; Supp Fig 1c: Arrows in Poly I:C and eRNA pairs). The figures in the main body of the manuscript should convey the reported findings without having to go to the supplementary data.

**Author Response:** I thank the reviewer for the suggestions. We have addressed the concern and necessary correction were made. We have merged the higher magnification images for figure 5 with the existing image data set Figure 5 (b-d). The arrow pointers of all the images have been checked and rectified of errors.

**Point: 3**

Similarly, the structures within Figure 5 (II) shows staining of CD11/CD18 and CD41 are still not discernible. Supplemental Figure 1d and 1e should be added to the main figure to bring additional clarity.

**Author Response:** I appreciate the reviewer for the comments. Accordingly, we have addressed the suggestion of the reviewer and merged the higher magnification images for figure 5 with the existing image data set Figure 5 (e-f).

**Point: 4:**

Thank you for providing the agarose gel images of the TLR3 siRNA knockdowns. Please add these to the supplemental data. The background should clearly state the purpose of the study.

**Author Response:** Acknowledging the comment by the reviewer, we have included the agarose gel image as Supplementary figure 2.
